# Effect of levels-of-processing on rates of forgetting

**DOI:** 10.3758/s13421-024-01599-4

**Published:** 2024-07-03

**Authors:** Nan Peng, Robert H. Logie, Sergio Della Sala

**Affiliations:** Human Cognitive Neuroscience, Department of Psychology, University of Edinburgh, 7 George Square, Edinburgh, EH8 9JZ UK

**Keywords:** Levels of processing, Long-term forgetting, Episodic memory, Encoding, Recognition

## Abstract

The levels-of-processing (LOP) framework, proposing that deep processing yields superior retention, has provided an important paradigm for memory research and a practical means of improving learning. However, the available levels-of-processing literature focuses on immediate memory performance. It is assumed within the LOP framework that deep processing will lead to slower forgetting than will shallow processing. However, it is unclear whether, or how, the initial level of processing affects the forgetting slopes over longer retention intervals. The present three experiments were designed to explore whether items encoded at qualitatively different LOP are forgotten at different rates. In the first two experiments, depth of processing was manipulated within-participants at encoding under deep and shallow conditions (semantic vs. rhyme judgement in Experiment 1; semantic vs. consonant-vowel pattern decision in Experiment 2). Recognition accuracy (*d* prime) was measured between-participants immediately after learning and at 30-min, 2-h, and 24-h delays. The third experiment employed a between-participants design, contrasting the rates of forgetting following semantic and phonological (rhyme) processing at immediate, 30-min, 2-h, and 6-h delays. Results from the three experiments consistently demonstrated a large effect size of levels of processing on immediate performance and a medium-to-large level effect size on delayed recognition, but crucially no LOP × delay group interaction. Analysis of the retention curves revealed no significant differences between the slopes of forgetting for deep and shallow processing. These results suggest that the rates of forgetting are independent of the qualitatively distinct encoding operations manipulated by levels of processing.

## Introduction

The study of forgetting dates back to the early work by Ebbinghaus (1885/1964). Primarily based on a verbal learning paradigm with non-meaningful syllables, Ebbinghaus plotted the maintenance of information (savings on re-learning) over time, often referred to as the forgetting curve. It is better described as the retention curve since the amount of retained information was portrayed. The classic curve presents a rapid drop shortly after learning, and then a slower decline that approaches an asymptote. Beyond non-meaningful syllables, this negatively accelerating pattern of forgetting has been observed across a wide range of learning materials (Rubin & Wenzel, 1996), such as verbal stimuli (Murre & Dros, 2015), faces and names (Bahrick et al., 1975), or real-world events (Meeter et al., 2005). Although a number of variables are known to affect acquisition, very few of these have reliable effects on delayed retention (Underwood, 1964). For instance, Slamecka and McElree (1983) showed that the slopes of forgetting for verbal lists remained constant between high and low degrees of learning (different number of repetitions during encoding) across varying learning materials (categorised words, paired associates, and sentences) and memory tests (free recall, cued recall, and recognition) over 1-day and 5-day delays. More recently, Rivera-Lares et al. (2022) reached the same conclusion by using a larger number of repetitions, varying the length of the retention intervals (30 s, 1 day, 3 days, and 1 week), and avoiding the confound of repeated retrieval of the same materials that can lead to relearning and slower forgetting (Baddeley et al., 2021; Roediger & Butler, 2011). This line of research offers evidence that forgetting rates tend to be independent of the initial degree of learning.

A possible interpretation for the parallel retention curves observed in the aforementioned studies is that a higher degree of learning boosted by repeated study or rote rehearsal does not lead to an elaborate or deep level of encoding, and thereby has little effect on the rates of forgetting. For instance, Sacripante et al. (2023) have shown that the gist of a prose passage appears to be forgotten at a slower rate than peripheral details. One possible account for this finding is that depth of processing during encoding (deeper for gist than for peripheral details) might have an impact on forgetting rates. Thus, different levels of processing during encoding might result in different forgetting rates. In this study, we aimed to investigate this possibility – namely, whether the depth of processing affects delayed retention and further modulates the rates of forgetting.

The levels-of-processing (LOP) framework, proposed by Craik and Lockhart (1972), conceptualises a view of human memory as a set of dynamic encoding and retrieval processes of mind and brain, as opposed to a variety of static structures or memory stores. It is argued that the memorability of information is a positive function of the depth or level of processing. Deep processing (e.g., conceptual, semantic or associative involvement) triggers a richer and more elaborate memory trace or neural network than shallow coding (e.g., perceptual, structural or phonological analysis). This greater richness or elaboration is assumed to support subsequent retrieval success. In addition, the deeply encoded information is more likely to be integrated with the person’s existing knowledge structures, which in turn serves as a more effective approach for reconstructive retrieval processes (Craik, 2002). Across ten experiments, Craik and Tulving (1975) demonstrated that deeper semantic analysis, compared with shallow processing during encoding, consistently yielded superior memory performance for verbal materials under both incidental and intentional learning conditions. Subsequently, a wealth of studies using different encoding tasks have provided substantial empirical support for the effects of levels of processing on free recall (Gardiner, 1974), cued-recall (Fisher & Craik, 1977; Moscovitch & Craik, 1976), recollection, and familiarity (Sheridan & Reingold, 2012). The advantage from deeper processing during encoding, when memory is tested after short retention intervals, has been reported for individual words (Seamon & Virostek, 1978), word pairs (Epstein et al., 1975), prose (Dixon & von Eye, 1984; Simon et al., 1982), pictures (Baddeley & Hitch, 2017; Marks, 1991), faces (Bower & Karlin, 1974; Warrington & Ackroyd, 1975), music (Mungan et al., 2011; Peretz et al., 1998), and olfactory stimuli (Royet et al., 2004), as well as across different age groups (Erber et al., 1980; Eysenck, 1975; Kheirzadeh & Pakzadian, 2016).

Although this facilitating level effect has been robustly reported in the immediate retrieval, very few studies have investigated this effect on delayed retention. According to the LOP framework, deeply processed information is assumed to be more available for retrieval from long-term memory, and is hypothesised to result in a flattened retention curve compared to shallow processing (Craik, 2002, 2021). However, this conjecture has not yet been thoroughly examined.

Epstein and Phillips (1976) claimed that items processed at deeper semantic levels decay more slowly than less deeply processed items. Although deeper processing yielded better retention performance at each timepoint for both related and unrelated pairs, a parallel decline was observed at 5- and 21-day delays, indicating an equal rate of forgetting between the two levels. However, floor performance (less than two out of 30 items recalled) on unrelated pairs and the effect of retrieval practice (recalling the same materials at different delays) masked the true retention change over longer delays. Nelson and Vining (1978) contrasted the effect of semantic and structural (syllable judgement) processing on long-term retention using unrelated individual words. Subsequent long-term retention performance, assessed by free recall after 4-day and 1-week delays, did not show a greater advantage for semantic processing over structural processing. The authors concluded that semantic versus structural processing has a potent effect on acquisition but little on long-term retention. In that study, the initial level of acquisition was equated between deep and shallow tasks by means of different repetitions of learning trials at encoding. As such, it is hard to know which of these two manipulations was responsible for the outcome. Similar forgetting characteristics were also reported in semantic and structural processing over a short delay (1–60 min) with a recognition test (McBride & Dosher, 1997; Experiment 4). Mixed findings arose from the analysis of variance and model comparisons using the power function. The interaction of delay and processing condition was not significant, suggesting that levels of processing did not influence the rates of forgetting. However, a significantly smaller slope estimate was observed following semantic processing relative to structural processing. This significant result was presumed to be contaminated by ceiling effects in the semantic condition. In contrast, a more recent study by Seiver et al. (2019) showed a steeper drop over 1- or 4-week delays on recalling word pairs under the deep (pleasantness rating) than the shallow (checking the letter “e” in words) condition across three experiments. As for previous studies, these findings are confounded by repeated testing of the same materials, and a floor effect at the longer delay.

The available LOP studies on long-term retention present divergent results and afford no satisfactory test of the assumption that deeper processing leads to a more durable and longer-lasting memory trace, or slower rates of forgetting than shallow processing. It seems plausible to postulate that well-learned information could resist forgetting more than poorly-learned material. However, there is still an empirical gap in our understanding of the relation between remembering and forgetting. It is theoretically important to test the hypothesis from the LOP framework regarding long-term retention. In so doing, the findings will add to understanding of the underlying mechanisms and characteristics of normal forgetting. For instance, if deep encoding flattens the retention curve relative to shallow encoding, it would imply selective consolidation of the newly learned information in favour of an elaborate and associative memory network. In contrast, if the shapes of forgetting were independent of the encoding operations, it would suggest that memory traces established through different encoding processes are equally susceptible to the processes of forgetting. If learning outcomes could be effectively upheld based on the initial degree of acquisition, methods that enhance immediate learning performance should also predict a substantial level of information maintenance. Moreover, insights we acquire from normal forgetting can contribute to how we define and characterise pathological forgetting, for example, accelerated long-term forgetting, found in individuals with amnesia who exhibit intact immediate memory performance, but experience accelerated forgetting over hours, days or weeks after learning (Butler et al., 2019; Hoefeijzers et al., 2019).

The present study was designed to address this issue by following the classic paradigm from Craik and Tulving’s (1975) study and exploring how the LOP affects delayed retention and the pattern of forgetting in episodic memory using individual words. Three issues are essential to consider. One is the testing effect, reflected in the enhancement of memory performance due to the effortful, repeated retrieval of information (Roediger & Karpicke, 2006; Roediger & Butler, 2011). Measuring long-term forgetting typically entails repeatedly retrieving learning materials at multiple timepoints. However, it has been reported by a meta-analytic review that the retrieval attempt for the same material can reliably affect the subsequent retention performance regardless of the format of memory tests (Rowland, 2014), thereby masking the power to detect changes in retention across different encoding conditions. To minimise the testing effect in the current study, different retention intervals (i.e., memory test at different delays) were arranged between participants. In addition, participants in each delay group were tested for recognition of half of the target words compared with distractors immediately after encoding, and identified the remaining subset of targets from a new set of distractors at the delayed test (Baddeley et al., 2021). A second issue concerns possible ceiling and floor effects. The ceiling effect refers to scores clustering toward the upper limit of the measure of performance, and the floor effect is the opposite. Performance approaching either scenario would no longer capture a relation between predictor and outcome variables and would distort the slopes of forgetting curves. To address the floor effect that often occurs over a longer delay, a recognition memory test and relatively short retention intervals (30 min, 2 h and 24 h) were used. Ceiling and floor effects are closely associated with the difficulty of the encoding task and the recognition test. Based on the results from Craik and Tulving’s (1975) experiments, two levels of processing induced respectively by rhyme and semantic orienting tasks were likely to generate a clear levels-of-processing effect while ensuring that performance for deep processing is not at ceiling and performance for shallow processing is above floor. Lastly, it has been demonstrated that instructions to learn have little effect on immediate and delayed memory performance (Craik & Tulving, 1975; Seiver et al., 2019; Walsh & Jenkins, 1973). However, intentional mnemonic strategies at encoding (e.g., rehearsing the items verbatim, extracting gist, organising the material into coherent structures, or forming visual images) are likely to affect the success of level manipulation and subsequent retention performance when additional time is allowed (McDaniel & Masson, 1977). As such, each encoding trial, including one target word and its corresponding orienting question, was paced at a 5-s presentation rate to reduce the likelihood that participants would develop effective strategies following intentional instructions.

## Experiment 1

### Methods

#### Study design

A 2 × 3 (LOP × delay) mixed study design, including one within-participants factor (the levels of processing – deep and shallow) and one between-participants variable (the time interval – 30 min, 2 h and 24 h), was employed to contrast recognition performance between deep and shallow conditions in individual words following the original LOP paradigm with two levels (phonological characteristic and semantic meaning) from Craik and Tulving (1975) Experiment 2.

#### Participants

An a priori power analysis, using G*Power 3.1, produced a minimum sample of 75 participants for three delay groups to achieve estimated effect sizes for LOP and delays (*f*_LOP_ = 0.30)^1^ in a two-way mixed analysis of variance (ANOVA), presuming a 5% significance level (two-sided) and a statistical power of 80%.

A total of 90 native English speakers (28 men) from the University and Prolific platform, with a mean age of 24.32 years (*SD* = 4.76, range = 18–35 years), were enrolled and randomly assigned to one of the delay groups (30-min, 2-h or 24-h delay) with an even group size (*n* = 30 for each group). Informed consent was obtained from all participants in accordance with the protocols approved by the School of Philosophy, Psychology and Language Sciences Research Ethics Committee. All participants were offered an honorarium of ₤5.

#### Materials

##### Stimuli

A total of 60 concrete English nouns of four to six letters, serving as the source of the target words, were extracted from Craik and Tulving’s Experiment 9 (1975). A pool of 200 distractors was selected from the MRC Psycholinguistic database (Wilson, 1988), matching the target words in length, frequency, concreteness, familiarity and imageability according to the SUBTLEX-UK database^2^ (van Heuven et al., 2014) and the MRC Psycholinguistic database (see Online Supplementary Materials (OSM)).

##### Encoding task

Encoding trials involved a series of level judgements, which were originated to tap either the phonological characteristic (Does the word rhyme with “shield”? field) or semantic meaning (Would the word fit the sentence? “The _____ grew larger in the wind.” flame) of the target words (Craik & Tulving, 1975). The encoding task included 40 level judgements with 40 target words presented in random order: 20 shallow and 20 deep questions with 10 ‘yes’ and 10 ‘no’ responses at each level. The target words, level judgements, and yes/no responses were rotated among individuals in a counterbalanced manner following the Latin square principle.

##### Recognition test

A total of 120 words, including 40 original targets and 80 distractors, served as the source of the immediate and delayed recognition tests. To minimise the effects of repeated retrieval, the immediate recognition test was composed of 20 targets (10 deep and 10 shallow encoded words) combined with 40 distractors, and the delayed test consisted of the remaining 20 targets and 40 new distractors, randomly intermixed.

#### Measures

Recognition memory performance was measured using a forced-choice Old/New recognition test. The hit rate (H) and the false alarm rate (FA) were calculated according to the Signal Detection Theory (Green & Swets, 1966; Macmillan & Creelman, 2004). The measure of discriminability employed *d* prime (*d*’, *z*-transformed hit rate minus *z*-transformed false alarm rate, *d*’ = *z*(H)-*z*(FA)), representing the ability of a participant to discriminate targets from distractors. The larger the *d* prime, the better the discrimination ability (Wixted, 2007). The extreme hit rates and false alarm rates (e.g., 0 or 1.0) were corrected using Hautus’s (1995) adjustments. Criterion location, (c, the negative value of half of the sum of *z*(H) and *z*(F), c = - $\frac{1}{2}$ (*z*(H) + *z*(FA)), was used as the detection of bias. It reflects a participant’s tendency or bias towards an ‘Old’ or ‘New’ option with a value of zero denoting an unbiased respondent. As the tendency to ‘Old’ increases, resulting in a higher hit rate and false alarm rate, the value approaches negative. If the value returns positive, it signifies the participant is more likely to respond to ‘New’ in the recognition task, leading to a lower hit rate and false alarm rate.

#### Procedure

Participants were invited to complete an online two-session experiment on the Testable platform (https://www.testable.org/) over two Microsoft Teams/Zoom video calls. During both sessions, each participant’s experiment window was shared with the researcher to prevent any potential misunderstanding of instructions and presentation issues, and to ensure that retest occurred after the retention interval assigned to each participant. The study began with the description of experiment information and the acquisition of informed consent. All participants were informed of the subsequent memory test and then assigned into three delay groups on the basis of different retention intervals (i.e., 30 min, 2 h and 24 h). Non-identifying demographic information, including gender and age, was first collected. A practice session was delivered to familiarise participants with the experimental procedure using different items from the formal sessions. Afterwards, each participant was expected to complete three programmed tasks (i.e., initial encoding, immediate recognition test, and delayed recognition test) independently using a keyboard and mouse. The entire study took up to 15 min.

**Fig. 1:**
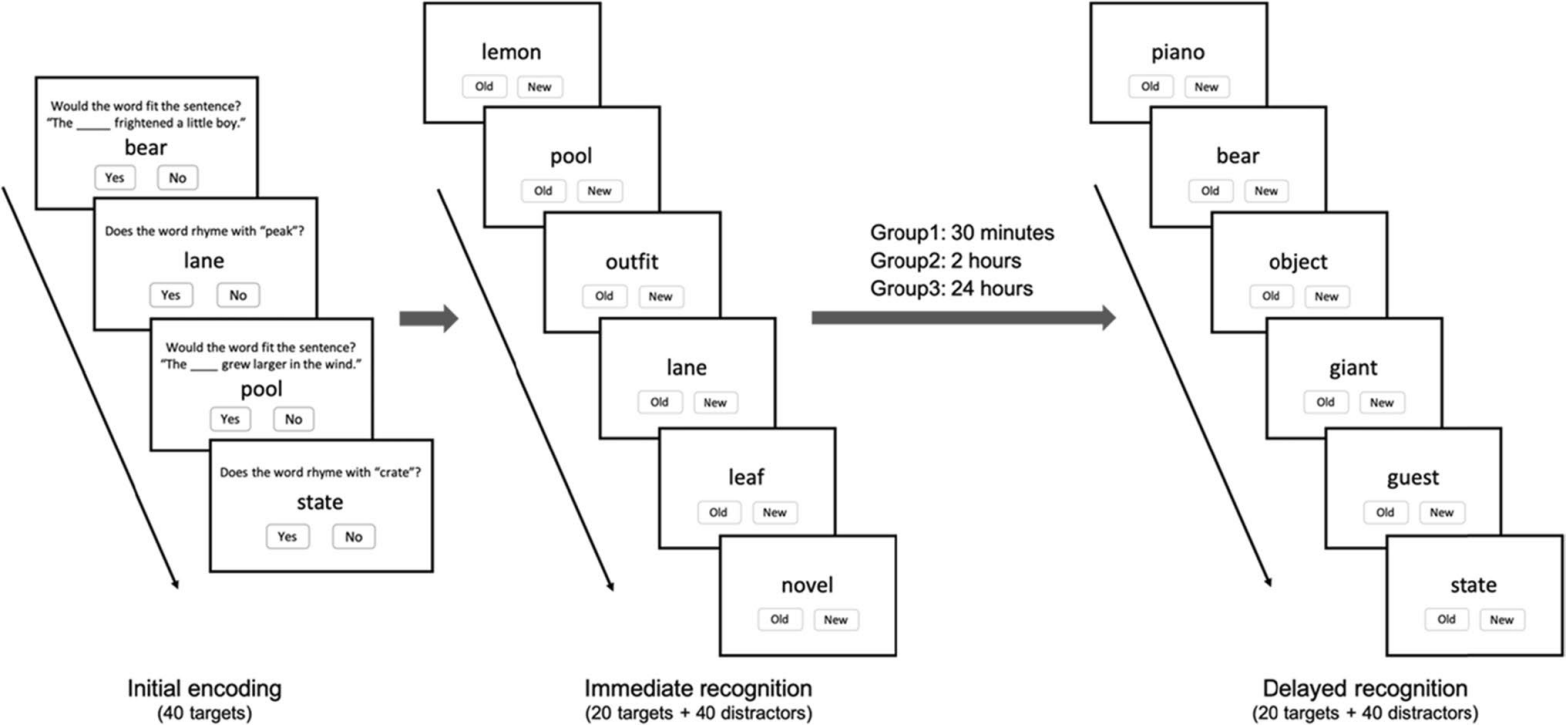
Experimental procedure of the encoding and retrieval trials in Experiment 1

During the initial encoding, the target word was presented in the centre of the screen with the question about the level judgement placed above and the Yes/No response options rested beneath the word. Participants were instructed to respond to each question by clicking the mouse on the Yes or No button as accurately and quickly as possible within a 5-s time limit. Next, an immediate recognition test was introduced to all participants using the Old/New forced choice. Participants were asked to react to the word by clicking ‘Old’ if it had been encountered in the encoding trial, otherwise clicking ‘New’. After the immediate retrieval, participants were informed that the second session, involving a delayed test administered over a Teams/Zoom call with the experimenter in the same manner as the immediate test, was expected 30 min later for Group 1 (*M* = 32.63 min, *SD* = 2.14 min), 2 h later for Group 2 (*M* = 1.95 h, *SD* = .09 h), and 24 h later for Group 3 (*M* = 23.95 h, *SD* = .17 h). The schematic procedure of the experiment is illustrated in Fig. 1.

#### Data analysis

All statistical analyses were performed in the R Statistical Environment (R Core Team, 2021, http://www.R-project.org/). Data preparation was conducted to detect missing values, performance outliers, and response bias. If the immediate recognition performance dropped to chance level (i.e., *d* prime = 0) or the variances from the hit rate or the false alarm rate fell beyond 2.5 standard deviations, the data were removed from the analyses. As such, four participants were removed from the further analyses due to poor recognition performance in the immediate test (two from the 2-h delay group and two from the 24-h delay group).

The response time (RT) and recognition performance^3^ (*d* prime) were compared using a two-way mixed ANOVA. The Type II sum of squares testing, based on the marginality principle, was run if the sample size in each group was unbalanced (Navarro, 2015). Post hoc comparisons using a Bonferroni-adjusted *p*-value were performed on any significant effects. Partial eta squared (${\eta }_{p}^{2}$) served as the measure of effect size for two main effects (level and time) and interaction with its conventional values of 0.01, 0.06, and 0.14 designating a small-, medium-, and large-sized effect, respectively (Perugini et al., 2018). To evaluate the strength of evidence in favour of the null or alternative hypothesis, a Bayesian version of ANOVA was performed using the R package *BayesFactor* with default priors (Morey & Rouder, 2022; Navarro, 2015). Following the conventions suggested by Kass and Raftery (1995), a Bayes factor (BF_10_ or BF_01_) in the range of 1–3, 3–20, 20–150, and > 150 represents the evidence for the alternative (or null) hypothesis as negligible, positive, strong, and very strong, respectively. We report BF_10_ when there was more support for the alternative than the null, and BF_01_ when the evidence favoured the null hypothesis.

The slopes of forgetting were assessed using likelihood-ratio tests to compare the fits of the nested models. Recognition accuracy on four retention intervals (immediate, 30-min, 2-h, and 24-h delays) was fit into four two-parameter retention functions by minimising squared deviations from its predictions: the linear ($y=a-bt$; Fisher & Radvansky, 2019), power ($y={a(t+1)}^{-b};$ Wixted & Ebbesen, 1991), exponential ($y={ae}^{-bt};$ White, 1985), and logarithmic function ($y=a-bln\left(t+1\right)$; Rubin & Wenzel, 1996). In these equations, *y* indicated the retention performance (*d* prime), *a* represented the initial degree of learning, *b* referred to the slope of the function, and *t* was the time. The goodness-of-fits of the models were assessed using the Akaike information criterion (*AIC*), the Bayes information criterion (*BIC*), and the Pearson’s coefficient (*R*^*2*^). The function that yielded the lowest *AIC/BIC* and the highest *R*^*2*^ value was selected to compare the slope parameters between deep and shallow conditions. For each level of encoding, the unconstrained model was specified allowing two parameters freely estimated. The slope parameter (*b*) from one condition was maintained to establish a constrained model for another. This constrained model was then compared with its unconstrained counterpart using the likelihood-ratio tests. A significant chi-square test (*p* < .05) would indicate that the slope of the retention function (*b*) varied statistically across different encoding conditions, suggesting the LOP has an effect on the rates of forgetting.

### Results

#### Encoding (response time (RT))

The average RTs for the shallow and deep tasks at encoding were 2,115 ms (*SE* = 69 ms) and 2,937 ms (*SE*, 106 ms), respectively. The two-way mixed ANOVA (Type II sum of squares) and Bayes factor analysis showed a significant main effect of levels on RT (after log transformation), *F* (1, 83) = 393.73, *p* < .001, ${\eta }_{p}^{2}$ = 0.22, BF_10_ > 150. Neither a main effect of delay groups, *F* (2, 83) = 0.18, *p* = .838, BF_01_ = 13.15, nor a LOP × delay group interaction, *F* (2, 83) = 2.20, *p* = .117, BF_01_ = 7.87, were found. The deep level of analysis required a longer RT relative to the shallow encoding.

#### Recognition performance

##### Immediate recognition

**Fig. 2:**
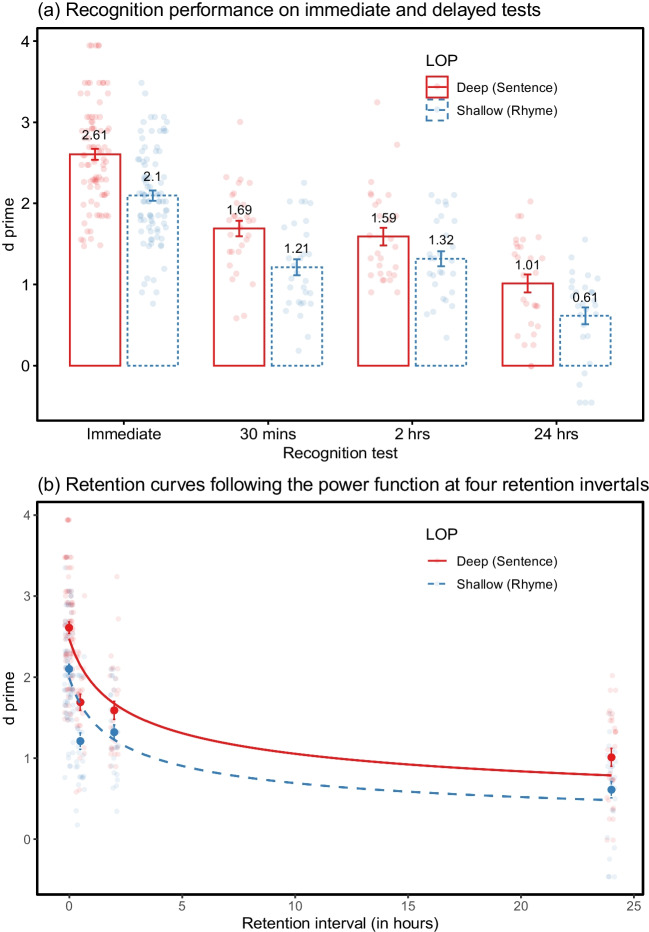
Means and standard errors of *d* primes at different delays under deep and shallow levels of processing (Experiment 1)

The *d* primes for shallow and deep encoding in the immediate recognition were 2.10 (*SE*, 0.06) and 2.61 (*SE*, 0.07), respectively (Table 1 and Fig. 2a). There was a significant main effect of LOP on *d* prime, *F* (1, 83) = 49.24, *p* < .001, ${\eta }_{p}^{2}$ = 0.37, BF_10_ > 150. Neither the main effect of delay groups, *F* (2, 83) = 0.75, *p* = .475, BF_01_ = 7.83, nor the LOP × delay group interaction, *F* (2, 83) = 0.57, *p* = .569, BF_01_ = 7.80, were found. The large-sized LOP effect on *d* prime in the immediate test suggest that the deep level of processing produces a better recognition accuracy and a higher degree of acquisition than shallow processing. Additionally, there were no significant differences between delay groups in their initial memory performance.

**Table 1:** Recognition performance (*d* prime, means and standard errors) on the immediate and delayed tests

Levels of processing	Retention intervals			
	Immediate	Delay 1	Delay 2	Delay 3
Experiment 1				
Deep (Sentence)	2.61 ± 0.07	1.69 ± 0.10	1.59 ± 0.11	1.01 ± 0.11
Shallow (Rhyme)	2.10 ± 0.06	1.21 ± 0.10	1.32 ± 0.09	0.61 ± 0.10
Experiment 2				
Deep (Sentence)	2.58 ± 0.07	1.67 ± 0.17	1.34 ± 0.11	0.93 ± 0.10
Shallow (Structure)	1.83 ± 0.09	1.02 ± 0.16	0.84 ± 0.11	0.59 ± 0.12
Experiment 3				
Deep (Sentence)	2.83 ± 0.08	1.63 ± 0.11	1.56 ± 0.13	1.43 ± 0.12
Shallow (Rhyme)	2.11 ± 0.06	1.22 ± 0.15	0.96 ± 0.12	0.66 ± 0.08

##### Delayed recognition

A significant main effect due to LOP on *d* prime, *F* (1, 83) = 44.90, *p* < .001, ${\eta }_{p}^{2}$= 0.35, BF_10_ > 150, was observed in the delayed recognition, indicating the LOP effect held over time. The main effect of delay groups was also significant, *F* (2, 83) = 16.99, *p* < .001, ${\eta }_{p}^{2}$ = 0.29, BF_10_ > 150. It showed that items were forgotten as time progressed. No LOP × delay group interaction was found, *F* (2, 83) = 1.10, *p* = .341, BF_01_ = 6.57. The change of recognition performance between deep and shallow conditions did not differ over three delay groups. Post hoc comparisons (Bonferroni adjusted *p*-value) over three retention intervals showed the recognition accuracy declined significantly from the 30-min to 24-h delay group, *t* (83) = 5.10, *p* < .001, Cohen’s *d* = 1.21, and from the 2-h to 24-h delay group, *t* (83) = 5.02, *p* < .001, Cohen’s *d* = 1.21, but not from the 30-min to 2-h delay group, *t* (83) = 0.01, *p* > .99.

#### The rates of forgetting

The recognition performance across four retention intervals was fit into four retention functions using least squares estimation (Table 2). The *AIC* and *BIC* measures were consistent with *R*^*2*^, in which lower AIC and BIC values were aligned with higher *R*^*2*^. The power function accounted for the highest proportion of variance under both deep and shallow conditions (*AIC*_*deep*_ = 338, *BIC*_*deep*_ = 348, *R*^*2*^_*deep*_ = .451; *AIC*_*shallow*_ = 314, *BIC*_*shallow*_ = 323, *R*^*2*^_*shallow*_ = .439). The logarithmic function also yielded a reasonable set of fits better than the exponential and linear functions. Figure 2b illustrates the retention curves following the power function under deep and shallow conditions. For both conditions, the power curves tended to overestimate performance at a short delay (30 min) and underestimate it at a longer delay (24 h).

**Table 2:** The goodness-of-fits of *d* prime between deep and shallow processing in the two-parameter version of linear, power, exponential and logarithmic functions

Retention functions	Experiment 1			Experiment 2			Experiment 3		
	*AIC*	*BIC*	*R*^*2*^	*AIC*	*BIC*	*R*^*2*^	*AIC*	*BIC*	*R*^*2*^
Linear: *y = a – bt*									
Deep processing	379	389	.303	322	331	.293	257	265	.302
Shallow processing	348	358	.315	331	340	.170	200	208	.432
Power: *y = a(t+1)*^*-b*^									
Deep processing	338	348	.451	280	289	.479	228	236	.454
Shallow processing	314	323	.439	306	315	.307	163	172	.591
Exponential: *y = ae*^*-bt*^									
Deep processing	375	385	.319	318	327	.314	250	258	.341
Shallow processing	343	353	.334	328	337	.186	183	191	.512
Log: *y = a – bln(t+1)*									
Deep processing	351	360	.409	295	304	.418	237	245	.411
Shallow processing	325	334	.402	316	325	.254	176	185	.540

To analyse the rates of forgetting, we restricted the model comparisons using the power function given its better estimation for the data. The unconstrained model for the shallow condition, ${y}_{shallow}= {a}_{1 shallow}{(t+1)}^{{-b}_{shallow}}$ was compared to its constrained counterpart with the same slope from the deep condition, ${y}_{shallow}= {a}_{2 shallow}{(t+1)}^{{-b}_{deep}}$. The likelihood-ratio test showed no significant difference between unconstrained and constrained models, χ^2^ (1) = 2.25, *p* = .134, suggesting the slopes of forgetting do not differ between deep and shallow processing (Table 3). This finding agrees with the null LOP × delay group interaction from the ANOVA model, implying that levels-of-processing have little effect on the rates of forgetting.

**Table 3:** Parameter estimates and model comparisons for *d* prime between unconstrained and constrained power models (Experiments 1 and 2)

Levels of processing	Parameter estimates		Model comparisons	
	*a* (SE)	*b* (SE)	χ^2^ value	*p* value
Experiment 1				
Deep (unconstrained)	2.47 (0.06)	0.36 (0.04)	/	/
Shallow (unconstrained)	1.99 (0.06)	0.44 (0.06)	2.25	.134
Shallow (constrained)	1.95 (0.05)	0.36		
Experiment 2				
Deep (unconstrained)	2.46 (0.07)	0.44 (0.06)	/	/
Shallow (unconstrained)	1.74 (0.08)	0.58 (0.11)	1.26	.262
Shallow (constrained)	1.69 (0.07)	0.44		

### Discussion

The forgetting pattern in Experiment 1 is negatively accelerating: the decline of recognition under both deep and shallow conditions was rapid shortly after initial encoding (within 30 min) and then slowed down over longer time intervals (from 30 min to 24 h). A significant main effect of groups on delayed recognition indicated the occurrence of forgetting. No significant difference in performance between 30 min and 2 h suggested a slower forgetting occurring after 30 min under the current experimental paradigm.

Experiment 1 succeeded in capturing a large-sized effect of LOP on immediate recognition accuracy. Deeper processing led to higher levels of performance at the immediate test than shallow processing, consistent with previous findings of LOP using verbal materials (Craik & Tulving, 1975; Hyde & Jenkins, 1969; Walsh & Jenkins, 1973). The average recognition accuracy between deep and shallow levels on immediate recognition achieved a discriminable starting point against which to assess subsequent forgetting. There were no significant differences in immediate recognition performance among the three delay groups, with all groups showing the LOP effect. The advantage of deep processing was maintained over time, supported by the significant main effect of LOP on delayed recognition. Notably, the analysis of the retention curves, captured by the power function, revealed that the depth of processing did not have a significant influence on the rates of forgetting, congruent with the null LOP × delay group interaction from the variance of analysis.

One possible confounding factor at encoding in Experiment 1 was the significant difference in RT between deep and shallow orienting tasks. Semantic processing required longer time to complete compared with the rhyme decision. Although it has been shown that longer encoding time is not necessarily associated with superior memory performance (Craik & Tulving, 1975), in Experiment 2, we attempted to increase the amount of time spent on shallow processing to explore whether time spent during encoding moderated the effect of LOP on the forgetting rates.

## Experiment 2

### Methods

#### Study design

A 2 × 3 (LOP × delay) mixed study design, including one within-participants factor (the levels of processing – perceptual structure and semantic meaning) and one between-participants variable (the retention interval – 30 min, 2 h and 24 h), was employed to contrast the recognition performance between deep and shallow conditions following the LOP paradigm from Experiment 5 (Craik & Tulving, 1975).

#### Participants

A total of 72 native English speakers^4^ (18 men), with a mean age of 22.12 years (*SD* = 2.93, range = 18–31 years), were enrolled from the University and randomly assigned to one of the delay groups (*n* = 24 for each group). Informed consent was obtained from all participants, and none had taken part in Experiment 1. All participants were given an honorarium of ₤5.

#### Materials, procedure and data analysis

The same 60 targets and 200 distractors as Experiment 1 were used as learning and testing materials. Level manipulations were arranged to tap either perceptual structure (Could the word be characterised as CVCCVC? singer. C = consonant; V = vowel.) or semantic meaning (Would the word fit the sentence? “The _____ frightened a little boy.” bear) of the target words. The procedure and data analysis remained the same as in Experiment 1. Participants were back for their second session, on average, 35.80 min (*SD* = 2.00 min) after the first session in the 30-min delay group, average 1.92 h (*SD* = .09 h) for the 2-h delay group, and 23.84 h on average (*SD* = .32 h) among the 24-h group.

### Results

#### Encoding (RT)

The average RTs for the shallow and deep tasks were 3,774 ms (*SE* = 106 ms) and 2,912 ms (*SE* = 82 ms), respectively. The two-way mixed ANOVA and Bayes factor analysis showed a significant main effect of LOP on RT (after log transformation), *F* (1, 66) = 119.35, *p* < .001, ${\eta }_{p}^{2}$ = 0.27, BF_10_ > 150. Neither a main effect of delay groups, *F* (2, 66) = 0.001, *p* = 1.00, BF_01_ = 14.51, nor a LOP × delay group interaction, *F* (2, 66) = 1.89, *p* = .160, BF_01_ = 4.81, were found. As predicted, the CV pattern judgement required significantly longer time than the semantic analysis.

#### Recognition performance

##### Immediate recognition

**Fig. 3:**
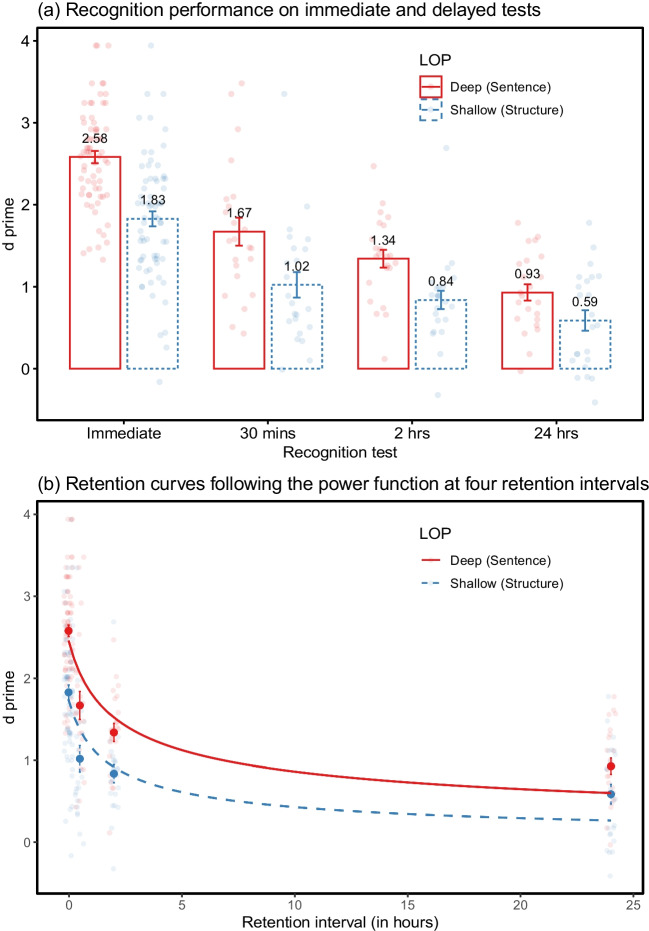
Means and standard errors of *d* primes at different retention intervals under deep and shallow levels of processing (Experiment 2)

The *d* primes for shallow and deep encoding in the immediate recognition were 1.83 (SE, 0.09) and 2.58 (SE, 0.07), respectively (Table 1 and Fig. 3a). A significant main effect due to LOP was observed, *F* (1, 66) = 87.43, *p* < .001, ${\eta }_{p}^{2}$ = 0.57, BF_10_ > 150. Neither a main effect of delay groups, *F* (2, 66) = 1.98, *p* = .146, BF_01_ = 2.07, nor a LOP × delay group interaction, *F* (2, 66) = 1.29, *p* = .283, BF_01_ = 5.39, was found. The large-sized level effects in the immediate recognition performance indicate that, though the shallow task required longer time to encode, the deep level of processing consistently presented a superior recognition accuracy and a higher degree of acquisition relative to shallow processing. Besides, there were no significant differences between delay groups in their initial memory performance.

##### Delayed recognition

A significant main effect of LOP, *F* (1, 66) = 34.60, *p* < .001, ${\eta }_{p}^{2}$ = 0.34, BF_10_ > 150, as well as a significant main effect of delay groups, *F* (2, 66) = 7.36, *p* < .001, ${\eta }_{p}^{2}$ = 0.18, BF_10_ = 103, were found in the delayed recognition. However, no LOP × delay group interaction was shown, *F* (2, 66) = 1.11, *p* = .336, BF_01_ = 5.76. The null interaction implies that the difference in retention performance between deep and shallow processing remains equivalent over three delay groups. Post hoc comparisons (Bonferroni-adjusted *p*-value) over three different delays indicated that *d* prime declined significantly from the 30-min to 24-h delay group, *t* (66) = 3.83, *p* < .001, Cohen’s *d* = 0.85, but not from the 30-min to 2-h, *t* (66) = 1.67, *p* = .298, nor from the 2-h to 24-h delay group, *t* (66) = 2.16, *p* = .104.

#### The rates of forgetting

Similar to Experiment 1, the highest proportion of variance was explained by the power function in both conditions (*AIC*_*deep*_ = 280, *BIC*_*deep*_ = 289, *R*^*2*^_*deep*_ = .479; *AIC*_*shallow*_ = 306, *BIC*_*shallow*_ = 315, *R*^*2*^_*shallow*_ = .307). The logarithmic function held the second place, performing better than the exponential and linear functions. Likewise, the estimated power curves predicted higher performance at short delays (30 min and 2 h) and lower performance at the 24-h delay (Fig. 3b). Unlike Experiment 1, the power curve fitted the deep condition more accurately than the shallow, presenting a differential estimation between semantic and structural tasks. The likelihood-ratio test indicated no significant differences between deep and shallow conditions in the slope parameters of the power function, χ^2^ (1) = 1.26, *p* = .262. This result is in line with the interaction analysis, suggesting that the rates of forgetting do not appear to be influenced by the qualitatively distinct encoding operations manipulated by the levels of processing.

### Discussion

Experiment 2 replicated the results from Experiment 1 with a shallow encoding task that took longer to perform than the deep encoding task. As before, the retention curve declined steeply within 30 min after initial encoding and then showed a much more gradual decline. In spite of more time spent on the consonant-vowel judgement, recognition accuracy was significantly higher under semantic than structural processing at the immediate retrieval, consistent with the previous findings from Craik and Tulving (1975). Additionally, three delay groups found no significant differences in their initial recognition. Like Experiment 1, the benefit of deep processing held over time with significant LOP effects on the delayed retrieval. However, the null model improvement and LOP × delay group interaction suggest again little effect of LOP on the rates of forgetting.

Given that the level manipulation was designated within participants, the false alarm rates between deep and shallow conditions were indistinguishable (see OSM). As a recognition test requires discrimination between old and novel stimuli, distractors provide valuable information on separating actual memory from response errors and judgement biases. The identical false alarm rates, therefore, provide little information on how depth of processing affects the capacity to distinguish distractors from targets. Moreover, studies have pointed out that false alarms were fewer when retrieving deeply encoded information compared with information following shallow processing (e.g., Jacoby, Shimizu, Daniels et al., 2005a; Jacoby, Shimizu, Velanova et al., 2005b). As such, it is important to investigate these findings in a between-participants design across short retention intervals to (1) further separate out deep and shallow distractors and (2) avoid the possibility of floor effects at a longer delay. This was the focus for Experiment 3.

## Experiment 3

### Methods

#### Study design

A 2 × 3 (LOP × delay) between-participants design was used to compare the recognition accuracy between deep (semantic) and shallow (phonological) processing across three retention intervals (i.e., 30 min, 2 h and 6 h).

#### Participants

A total of 120 native English speakers^5^ (37 men), with a mean age of 24.05 years (*SD* = 5.39, range = 18–35 years), were enrolled from the University and Prolific platform and randomly assigned to one of the delay groups (*n* = 20 for each group). Informed consent was obtained from all participants, and none had taken part in Experiment 1 or Experiment 2. All participants were given an honorarium of ₤3 or course credit.

#### Materials

The same 60 targets and 200 distractors as Experiment 1 were used as learning and testing materials. The encoding task included 40 level judgements at either deep or shallow processing with 40 target words presented in random order. In the shallow condition, the targets were processed according to their phonological features (Does the word rhyme with stock? rock), whereas in the deep condition, the semantic meaning of the targets was processed (Would the word fit the sentence? “I tore my jeans on the _____.” fence). The immediate and delayed recognition tests were structured the same as in Experiment 1. After the first session, participants in the 30-min delay groups returned for their second session, averaging 35 min (*SD* = 2.52 minutes) in the deep condition and 34 mins (*SD* = 2.26 min) in the shallow condition. Those in the 2-h groups, following deep and shallow conditions, started the delayed session 2.01 h (*SD* = .15 h) and 2.01 h (*SD* = .06 h) after the completion of the first session, respectively. Participants in the 6-h delays, under the deep and shallow conditions, re-joined the second session after 5.94 h (*SD* = .22 h) and 6.02 h (*SD* = .27 h), respectively.

#### Procedure and data analysis

The procedure remained the same as Experiment 1 except that levels were manipulated between participants and the delayed recognition test was delivered between participants at 30 min, 2 h and 6 h after the immediate test. The RT and *d* prime were computed and analysed using the two-way factorial ANOVA and Bayes factor analysis. The Type II sum of squares testing was run if the sample size in each group was unbalanced. Tukey’s “Honestly Significant Difference (HSD)” test was performed as post hoc comparisons to control the Type I error rates (Navarro, 2015). The slopes of forgetting were contrasted between deep and shallow conditions using the likelihood-ratio test. Six participants were excluded from the statistical analyses due to their poor recognition performance in the immediate test (four from the rhyme condition and two from the semantic group).

### Results

#### Encoding (RT)

The average RTs for shallow and deep encoding were 1,778 ms (*SE* = 58 ms) and 2,711 ms (*SE*, 81 ms), respectively. The two-way factorial ANOVA (Type II sum of squares) and Bayes factor analysis showed a significant main effect of LOP on RT (after log transformation), *F* (1, 108) = 91.63, *p* < .001, ${\eta }_{p}^{2}$ = 0.46, BF_10_ > 150. Neither a main effect of delay groups, *F* (2, 108) = 0.43, *p* = .649, BF_01_ = 8.75, nor a LOP × delay group interaction, *F* (2, 108) = 0.81, *p* = .446, BF_01_ = 4.04, were found. The semantic analysis required more time than rhyme judgement.

#### Recognition performance

##### Immediate recognition

**Fig. 4:**
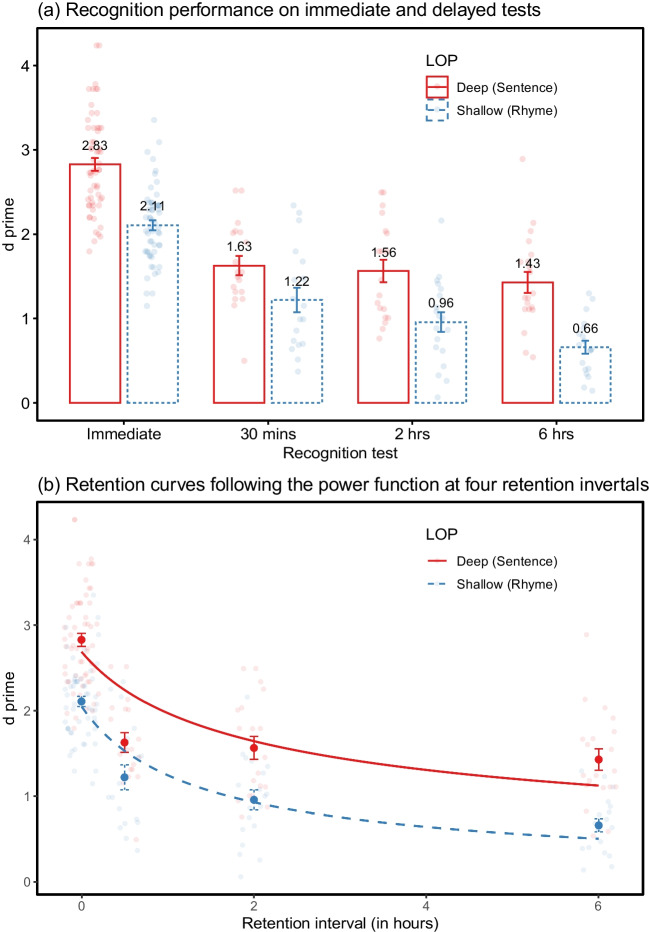
Means and standard errors of *d* primes at different retention intervals under deep and shallow levels of processing (Experiment 3)

The *d* primes for shallow and deep encoding in the immediate recognition were 2.11 (*SE*, 0.06) and 2.83 (*SE*, 0.08), respectively (Table 1 and Fig. 4a). There was a significant main effect of LOP on *d* prime, *F* (1, 108) = 55.45, *p* < .001, ${\eta }_{p}^{2}$= 0.34, BF_10_ > 150. Neither a main effect of delay groups, *F* (2, 108) = 2.02, *p* = .138, BF_01_ = 2.41, nor a LOP × delay group interaction, *F* (2, 108) = 1.16, *p* = .318, BF_01_ = 3.23, was found. The deep encoding yielded a higher recognition accuracy relative to shallow processing. There were no significant differences between three delay groups in their initial memory performance.

##### Delayed recognition

A significant main effect of LOP on *d* prime was found in the delayed recognition, *F* (1, 108) = 36.82, *p* < .001, ${\eta }_{p}^{2}$ = 0.25, BF_10_ > 150, indicating the level effect remained over time. The main effect of delay groups, *F* (2, 108) = 4.88, *p* = .009, ${\eta }_{p}^{2}$ = 0.08, BF_10_ = 4.15, was also significant, suggesting retention performance declined with the passage of time. Again, no LOP × delay group interaction was found, *F* (2, 108) = 1.12, *p* = .331, BF_01_ = 3.21. Further post hoc comparisons, by means of Tukey’s HSD correction to adjust *p*, indicated no significant changes from 30 min to 2 h (*p* = .347) or from 2 h to 6 h (*p* = .202), but a significant difference between 30-min and 6-h delays (*p* = .007).

#### The rates of forgetting

Like Experiments 1 and 2, the power function accounted for the highest proportion of variance for both deep and shallow conditions among four retention functions (*AIC*_*deep*_ = 228, *BIC*_*deep*_ = 236, *R*^*2*^_*deep*_ = .454; *AIC*_*shallow*_ = 163, *BIC*_*shallow*_ = 172, *R*^*2*^_*shallow*_ = .591), followed by the logarithmic function with comparable fits (*AIC*_*deep*_ = 237, *BIC*_*deep*_ = 245, *R*^*2*^_*deep*_ = .411; *AIC*_*shallow*_ = 176, *BIC*_*shallow*_ = 185, *R*^*2*^_*shallow*_ = .540). Similarly, as illustrated in Fig. 4b, the power curves predicted higher accuracy at the 30-min delay and lower accuracy at the 6-h delay than the actual recognition performance. In contrast to Experiments 1 and 2, the power function yielded a better estimation in the phonological than the semantic task. We discuss this differential fitting between deep and shallow tasks in the *General discussion*. The likelihood-ratio test indicated a significant change between unconstrained and constrained power models, χ^2^ (1) = 13.48, *p* < .001, suggesting forgetting rates differ between deep and shallow processing (Table 4). A further exploration of the forgetting slopes in the runner-up logarithmic function, however, revealed a null difference between deep and shallow processing in the slope parameter, χ^2^ (1) = 0.10, *p* = .747, agreeing with the null interaction from the analysis of variance. This discrepancy is discussed in the next section.

**Table 4:** Parameter estimates and model comparisons for *d* prime between unconstrained and constrained power and logarithmic models (Experiment 3)

Levels of processing	Parameter estimates		Model comparisons	
	*a* (SE)	*b* (SE)	χ^2^ value	*p* value
Power: *y = a(t+1)*^*-b*^				
Deep (unconstrained)	2.69 (0.08)	0.45 (0.05)	/	/
Shallow (unconstrained)	2.05 (0.06)	0.72 (0.08)	13.48	< .001
Shallow (constrained)	1.93 (0.06)	0.45		
Log: *y = a - bln(t+1)*				
Deep (unconstrained)	2.62 (0.08)	0.75 (0.08)	/	/
Shallow (unconstrained)	1.97 (0.06)	0.77 (0.07)	0.10	.747
Shallow (constrained)	1.96 (0.05)	0.75		

### Discussion

In a between-participants design, similar to Experiments 1 and 2, the large-sized LOP effect was acquired at immediate recognition and there were no significant differences across delay groups in their initial performance. Significant LOP effects carried over at delayed retrieval, but no LOP × delay group interaction was found. This result was supported by the analysis of forgetting rates following the logarithmic function but not in the power function.

This discrepancy emerges likely because the intercept parameter (*a*), indicating the initial memory performance, is dependent on the slope parameter (*b*) in the power function mathematically but independent in the logarithmic function or in the ANOVA model. In this case, the slope component in the power function would be influenced jointly by both the intercept and time variables via its multiplicative nature. It is potentially confounded where the intercept (i.e., the initial level of performance) is the factor of interest and manipulated at encoding (Della Sala et al., 2024). This confound would be more evident when the time interval tended to be short in Experiment 3. It can also explain the same disagreement between the power-fit and ANVOA approach observed in McBride and Dosher’s (1997) study, in which the ceiling performance from semantic orienting condition noticeably contaminated the slope fit within 1- to 60-min delays. As such, the conclusion in Experiment 3 is drawn based on the factorial ANOVA with the Bayes factor analysis and the logarithmic model comparisons.

Taken together, the results from three experiments collectively indicated that the rates of forgetting are independent of the qualitatively distinct encoding operations manipulated by levels of processing.

## General discussion

The present series of experiments extended the effect of LOP from immediate to delayed memory performance, exploring the critical question of whether the depth of processing at encoding affects the delayed retention and further influences forgetting rates in verbal materials. Different levels of encoding (i.e., structural, phonological and semantic processing) were manipulated across three experiments. Recognition accuracy was assessed immediately and at three delayed intervals (30 min, 2 h, and 24 h in Experiments 1 and 2; 30 min, 2 h and 6 h in Experiment 3). Delayed retention was contrasted through the interaction between the retention interval and the recognition performance under deep and shallow conditions. Forgetting rates were analysed between the restricted and full models in multiple retention functions.

As predicted by the LOP framework, deeper processing yielded superior recognition performance on *d* prime than shallow processing at immediate retrieval with an average effect size based on partial η^2^ of 0.43. This advantage in retention was maintained at delayed retrieval with a mean partial η^2^ of 0.31. Importantly, the LOP × delay group interaction did not approach significance and the forgetting rates across varying depths of processing appear to be the same. Compared with previous studies, the current design controlled the confounds from the repeated study at encoding, minimised the testing effect at retrieval, and effectively manipulated performance off the ceiling at the first test and above the floor at a longer delay. The analysis included both the ANOVA model with Bayes factors and the evaluation and comparison for the retention curves. Our results, consistent with Nelson and Vining’s (1978) observation in free recall and McBride and Dosher’s (1997) Experiment 3 in cued recall, suggest an equivalent forgetting rates between deep and shallow processing in recognition memory. Extending the extant literature on initial learning and forgetting (e.g., Meeter et al., 2005; Rivera-Lares et al., 2022; Slamecka & McElree, 1983), using a different paradigm for assessment memory, our findings add support to the conclusion that forgetting rates are not dependent on initial levels of learning.

The retention curves in the current study followed a negatively accelerating pattern with fast forgetting occurring shortly after learning and then slowing down with the passage of time. The estimated power curves apparently overestimated recognition performance at a short delay (30 min) and underestimated it at longer delays (6 h and 24 h) across all three experiments. This observation is consistent with McBride and Dosher’s (1997) Experiments 3 and 4, where it was assumed that one single function had difficulty in simultaneously capturing the rapid early drop and the later slow decline. Meanwhile, the differential fits in each function between deep and shallow tasks have raised a long-lasting question regarding which function provides the closest mathematical fit to the retention curve; more importantly, whether one single function can adequately characterise the maintenance of diverse learning materials over different time periods. The form of forgetting has been discussed among several candidates, such as logarithmic (Ebbinghaus, 1885/1964), power (Wixted & Ebbesen, 1991), exponential (White, 1985), exponential-power (Wickelgren, 1972, 1974), hyperbolic (Harnett et al., 1984), and linear function (Fisher & Radvansky, 2019). For instance, Rubin and Wenzel (1996) fitted 105 different two-parameter functions to a sample of 210 published reports and concluded that the logarithmic, power, exponential in the square root of time, and hyperbola in the square root of time fitted most datasets equally well. However, a recent challenge has been made to question the generalisation of one function to all patterns of memory change over time. The retrieval accuracy from fragmented traces (RAFT) model, for instance, suggests that memory retrieval during different periods of time has different retention and forgetting characteristics (Radvansky et al., 2022). In some scenarios, such as higher degrees of learning and meaningful materials, linear forgetting can be observed (Fisher & Radvansky, 2019). In the present study, the unsatisfactory predictions from one retention function and the discrepancy in model fits between deep and shallow memory traces are in line with this idea, and underscored the limitation of over-reliance on one single function to capture the entire trajectory of memory change over time.

Theoretical accounts for the independence of forgetting rates from levels of processing, or more broadly speaking, from the initial degree of learning, remain unclear, as neither the LOP framework nor existing cognitive theories of forgetting have yet provided a satisfactory explanation for this counterintuitive phenomenon. It has been assumed that the superiority of acquisition under deeper processing results from the richer and more elaborate memory traces triggered by broad cognitive involvement of semantics and association. Subsequent retrieval should be more likely to succeed as previously broader activations or connections scaffold greater accessibility or more possible links to appropriate memory cues (Craik, 2002, 2021; Craik & Lockhart, 1972). This account suits the observations reported in the existing literature and the present experiments in which the effect of LOP is held as time progresses. However, the size of this advantage was constant over time, which is incompatible with the assumptions of the LOP approach. Given the uniform shape of retention curves under deep and shallow conditions, it is hard to argue that deeper processing is more resistant to factors that facilitate forgetting, such as slower trace decay, reduced interference from competing items, or low impact of changes in context between encoding and retrieval.

A possible theoretical candidate, in line with an adaptive view of active forgetting, is that neurobiology supports a process that functions to encourage information loss, instead of information maintenance, through altering the state of memory or its contextual cues (Anderson & Hulbert, 2021; Davis & Zhong, 2017; Hardt et al., 2013; Nørby, 2015; Wixted, 2004). This intrinsic forgetting might act as a default state of the brain, constantly and indiscriminately leading to the inaccessibility of recently formed memory traces, no matter how they are initially established. This process is more likely to occur during the initial phases of consolidation and reconsolidation when hippocampal traces are more prone to modulation (Dudai, 2004; Squire, 1992). The natural forgetting, as an active and adaptive processing, continually reverses the spatial-temporal contextual scaffolds linking to the content representations of experimental episodes (Moscovitch et al., 2016; Nadel, 2008; Teyler & Rudy, 2007). This adaptive function would allow individuals to maintain greater flexibility and functionality of the memory systems, preventing information overload and preparing for the new learning. External or internal factors that prompt forgetting, such as retroactive and proactive interference or cue overload, may collectively but differentially contribute at distinct stages of information processing (i.e., encoding, maintenance and retrieval), depending on the nature and structure of learning materials. Aligned with this idea, richer and more elaborate schematic representations are activated following a deep level of processing; superior immediate retrieval benefits from this broad connection. However, the equal rates of forgetting between the deep and shallow condition observed in the present study may reflect that the speed of contextual trace reversal remains constant and indiscriminate.

The findings from the present experiments have several significant theoretical and practical implications. First, it upholds the importance of the LOP framework for improving learning outcomes, given that large LOP effect sizes have been observed for both immediate and delayed memory tests. This indicates that the initial level effect maintains over time. The equal forgetting rates for deep and shallow memory traces, moreover, might in turn imply a possible way of how distinctly they are initially established during encoding. The elaborate memory traces triggered by deep processing may indicate broader networking or association instead of stronger memory strength, because the latter is expected to result in a slower forgetting rate following deep encoding, and this might not apply to the former. The former interpretation is also aligned with findings from neuroimaging studies in which the encoding of deeply studied words correlated with broader neural recruitments relative to those words encoded in a shallow task (Baker et al., 2001; Fletcher et al., 2003; Otten et al., 2001; Wagner et al., 1998). Second, efforts have been made to identify various factors determining remembering success, for instance, the total time hypothesis, the practice effect, and levels of processing approach (Baddeley et al., 2015; Cooper & Pantle, 1967; Craik & Tulving, 1975; Ericsson, 1993). However, emerging evidence, including from the present experiments, has revealed that the factors facilitating acquisition seem to have little effect on the rates of forgetting. For educators and learners, it would imply that additional efforts should be made in retaining learning outcomes, such as employing strategies like distributed learning or retrieval-based learning to counteract the natural forces of forgetting.

Attempts could be also made in future studies by building on the present experimental paradigm and results. For instance, the to-be-remembered materials used in the current study were unrelated words. Like the Ebbinghaus tradition, highly constrained materials, such as non-meaningful syllables or individual items, intentionally attempt to rule out the relation or structure between items. This approach helps to minimise the influence of existing knowledge and our use of different subsets of unrelated words for testing at each retention interval reduces the impact of priming effects across delay intervals (Baddeley et al., 2019, 2021). However, it limits the generalisation of findings to the situations outside the laboratory. Although the individual words under semantic encoding were processed deeply in the current study, they were less likely to be organised into a structure as coherently as an event or a prose used in Sacripante et al. (2023). The differences in the type of learning materials may account for the diverging findings in forgetting rates observed in gist and peripheral memory (Helm & Reyna, 2023). As such, in future studies, a naturalistic approach pioneered by Frederick Bartlett (1932) could complement this limitation by employing integrated learning materials (e.g., narratives, news, video clips) with greater ecological validity than empirical observations based on more constrained materials such as words or word pairs. Moreover, retention performance could be further assessed using alternative forced choice (AFC) or receiver operating characteristics (ROCs) analysis to tackle the response bias in recognition (Brady et al., 2022). In addition, relatively short retention intervals (no more than 24 h) were chosen to avoid the complications from interpreting floor effects in delayed recognition. The forgetting curve portrays a quantitative relationship between retention performance and time. Therefore, providing that floor effects are avoided, more and longer time intervals could be investigated for greater precision of forgetting functions plotted from larger data sets, hopefully yielding deeper insights into the characteristics of forgetting over time.

In conclusion, our experiments add new knowledge to the understanding of the effects of long-term forgetting on levels of processing during encoding. We find superior immediate memory acquisition following deep over shallow levels of encoding, and this facilitating effect of deep encoding holds with the passage of time. Crucially, the rates of forgetting are independent of the degree of learning manipulated by the depth of encoding.

## Data Availability

The learning materials and behavioural data for the current study are available in a public repository at https://osf.io/phm7t/.

## Footnotes

1 The size of the LOP effect was estimated according to a recent LOP literature (Baddeley & Hitch, 2017), in which the mean effect size of LOP in verbal stimuli was from 0.6 to 1.4 based on Cohen’s *d*. The present study employed the lowest size (Cohen’s *d* = 0.6) from the aforementioned study.

2 The SUBTLEX-UK is a recently developed, subtitle-based word frequency database for British English, collected from nine BBC channels broadcast over a period from January 2010 to December 2012. The SUBTLEX-UK employs a standardised frequency measure, the *Zipf* scale ranging from 1 to 7. Words with *Zipf* values lower than 3 are categorised as low-frequency words. *Zipf* values of 4 and higher indicate high-frequency of usage.

3 3 Recognition performance was analysed based on *d* prime. Given similar findings, hit rates and false alarms are reported in the Online Supplementary Materials (OSM).

4 4 An a priori power analysis using G*Power 3.1 estimated a minimum sample of 18 participants for three groups to reach the effect size of LOP (*f*_level_ = 0.73, partial η^2^ = 0.35) acquired from Experiment 1, in a two-way mixed ANOVA, presuming a 5% significance level (two-sided) and a statistical power of 80%.

5 5 An a priori power analysis using R package *pwr2*, estimated a minimum sample of 30 participants for six groups to acquire the effect sizes for levels and time (*f*_level_ = 0.73, *f*_time_ = 0.64), acquired in Experiment 1, in a two-way factorial ANOVA, assuming a 5% significance level (two-sided) and a statistical power of 80%. Conservatively, medium-to-large effect sizes (*f*_level_ = 0.30, *f*_time_ = 0.30) were taken to estimate the minimum sample size (N = 114) for Experiment 3.

## Code availability

The analysis code is available in a public repository at https://osf.io/phm7t/.
